# Stillbirth Among Women Prescribed Nicotine Replacement Therapy in Pregnancy: Analysis of a Large UK Pregnancy Cohort

**DOI:** 10.1093/ntr/nty019

**Published:** 2018-01-31

**Authors:** Nafeesa N Dhalwani, Lisa Szatkowski, Tim Coleman, Linda Fiaschi, Laila J Tata

**Affiliations:** 1 Division of Epidemiology and Public Health, University of Nottingham, Clinical Sciences Building, Nottingham City Hospital, Nottingham, United Kingdom; 2 Division of Primary Care, University of Nottingham, Queen’s Medical Centre, Nottingham, United Kingdom; 3 Diabetes Research Centre, University of Leicester, Leicester General Hospital, Leicester, United Kingdom

## Abstract

**Introduction:**

We aimed to compare risk of stillbirth between maternal smokers and those prescribed nicotine replacement therapy (NRT) during pregnancy.

**Aims and Methods:**

We conducted a cross-sectional analysis on a pregnancy cohort of 220,630 singleton pregnancies ending in live or stillbirth between 2001 and 2012 from The Health Improvement Network UK general practice database. Women were categorized into three groups: NRT (prescribed during pregnancy or 1 month before conception); smokers; and controls (nonsmokers without a pregnancy NRT prescription). We calculated Odds ratios (OR) and corresponding 95% confidence intervals (CI) for stillbirth in the NRT group and smokers compared to controls.

**Results:**

A total of 805 pregnancies ended in stillbirth (3.6/1000 births). Absolute risks of stillbirth in NRT and smoker groups were both 5/1000 births compared with 3.5/1000 births in the control group. Compared with the control group, the adjusted odds of stillbirth in the NRT group was not statistically significant (OR = 1.35, 95% CI 0.91 to 2.00), although it was similar in magnitude to that in the smokers group (OR = 1.41, 95% CI 1.13 to 1.77).

**Conclusions:**

We found no evidence of a statistically significant association between being prescribed NRT during pregnancy and odds of stillbirth compared with nonsmoking women. Although our study had much larger numbers than any previously, an even larger study with biochemically validated smoking outcome data and close monitoring of NRT use throughout pregnancy is required to exclude effects on findings of potential exposure misclassification.

## Introduction

Maternal smoking during pregnancy increases the risk of several adverse birth outcomes including stillbirth. A meta-analysis of four studies from Australia, Sweden, Canada, and the United States found maternal smoking to increase the risk of stillbirth by 36%.^1^ Despite this, 12% of pregnant women in the United Kingdom,^2^ 13% in the United States,^3^ and 15% in Australia^4^ still smoke during pregnancy. Therefore, reducing smoking during pregnancy is a global public health priority. Pharmacotherapy, specifically the use of nicotine replacement therapy (NRT) is being adopted in several national guidelines for supporting pregnant smokers to quit, based on the notion that NRT is probably safer than smoking.^5–7^ In the United Kingdom, approximately 11% of pregnant smokers are prescribed NRT in primary care,^8^ despite a lack of evidence concerning its safety in pregnancy.^9^ Evidence specifically in relation to stillbirth is limited to one population-based study using the Danish National Birth Cohort (DNBC) which found no increased risk of stillbirth associated with NRT use in the first 27 weeks of pregnancy (hazard ratio [HR] = 0.57, 95% confidence interval [CI] 0.28 to 1.16 compared with those who did not use NRT and did not smoke). Information on NRT use and smoking status in the DNBC was ascertained up to the time of interview, at approximately 17 weeks of gestation, so it is possible that women’s exposure status may have changed during the second or third trimesters. A meta-analysis of four randomized controlled trials assessing stillbirth as a secondary outcome reported a raised but not significant pooled risk ratio of 1.24 (95% CI 0.54 to 2.84) for NRT use compared with placebo.^10^ However, randomized controlled trials assessing effectiveness of NRT patches and gum for quitting do not provide such safety evidence as they have inadequate power to assess rare outcomes.^11–13^

In light of limited safety evidence, in its recent international guidelines on prevention and management of tobacco use in pregnancy, the World Health Organization (WHO) recognized an urgent need for more research into the effects of NRT on pregnant women and the fetus, along with its efficacy.^14^ Therefore, using a large population-based pregnancy cohort, we investigated whether NRT prescribed in UK primary care is associated with stillbirth, compared with pregnant women recorded as smokers not prescribed NRT and a nonsmoker control group.

## Methods

### Data Source and Study Population

The Health Improvement Network (THIN) is a UK database of anonymized electronic primary care records, including sociodemographic information, diagnoses, prescriptions, and investigations. The validity of recorded diagnoses and prescriptions is high,^15^ and THIN has been previously validated for its recorded population prevalence of smoking at a national level for both the general population^16^ and pregnant women.^17^ Furthermore, fertility rates in THIN are highly comparable to national fertility rates.^18^ At the time of this study, THIN contained longitudinal prospectively collected data from 570 general practices across the United Kingdom, covering 6% of the UK population.^19^ We created our study population extracting all the singleton pregnancies with deliveries between 2000 and 2013.

### Exposure

All pregnant women with a recorded prescription for NRT during pregnancy in their primary care records were identified, using Multilex drug codes for all formulations available in the United Kingdom according to the British National Formulary (BNF),^20^ and classified as the NRT group, provided they did not have a current smoking Read code following the NRT prescription. Women with a prescription for NRT in the 4 weeks before conception were also included in the NRT group because it is likely that the medication could have been consumed during the early stages of pregnancy. Smoking status during pregnancy was determined from a previously validated algorithm using smoking status Read codes.^16,17^ Using this algorithm, we categorized women as smokers (those recorded as smokers at any point from conception until delivery) or controls (those exclusively recorded as nonsmokers at any point from conception until delivery). In 2004, the introduction of the Quality and Outcomes Framework (QOF) brought several pay-for-performance targets to primary care, including the electronic recording of smoking and smoking cessation advice. Since these data are recorded in routine primary care where repeat recordings for ex- and never-smokers are not required in certain scenarios, use of these QOF rules further facilitated identification of controls.^21^ Firstly, if there was no smoking status record during pregnancy, but women were recorded as never-smokers at any time during their active registration period when they were over 25 years of age, they were included in the control group. Secondly, if a woman did not have a smoking status record during pregnancy but was recorded as an ex-smoker for three consecutive years before pregnancy, we categorized her as an ex-smoker and she was thus included in the control group. After considering these QOF rules, all remaining women with missing smoking status were excluded from the study. More details on this approach of defining nonsmoking controls have been previously published.^9^

### Outcome

Stillbirth was defined as a baby born with no signs of life at or after 28 weeks of gestation, in accordance with the WHO definition.^22^ Information on the following potential confounders was also extracted due to known associations between these factors/conditions or their treatments and both stillbirth and maternal smoking:^23–31^ women’s age at conception; socioeconomic deprivation (quintile of the Townsend Index of deprivation);^32^ pre-pregnancy body mass index; and recorded diagnoses of medical conditions before or during pregnancy (hypertension, epilepsy, diabetes, asthma, and mental illness including depression, anxiety, bipolar disorder, schizophrenia, and other psychoses). All code lists are available from the authors on request.

### Statistical Analysis

The absolute risk of stillbirth was calculated as the total number of stillbirths divided by the number of stillbirths and live births combined. This was estimated for the entire population as w**e**ll as each exposure group (ie, NRT group, smokers, and controls). We used logistic regression to compute odds ratios (OR) and corresponding 95% CIs for stillbirth for the NRT group and smokers, compared with the control group. All potential confounders that had a statistically significant association (*p* < .05) with the exposure and the outcome in chi-squared tests were included in the final model. Some women in the study period had more than one pregnancy, and therefore, we used generalized estimating equations with an exchangeable correlation structure to take potential correlation between pregnancies into account.^33^ The reference group was then changed to smokers, and the ORs and corresponding 95% CIs were recalculated. All analysis was conducted in Stata MP 12 (Stata Corp., College Station, TX).

## Results

### Baseline Characteristics

The study population included 220 630 singleton pregnancies delivered from 2001 to 2012, of which 805 ended in stillbirth, a prevalence of 3.6/1000 births. Table 1 presents the baseline characteristics of mothers overall and by birth outcome. Pregnancies that were conceived at later maternal ages (≥35 years) resulted in a higher prevalence of stillbirth. Diabetes was also more common in pregnancies that ended in stillbirth compared with live births (5.0% vs. 3.2%). The distribution of socioeconomic status and the prevalence of other chronic illnesses like asthma, hypertension, and mental illness were comparable in women with stillbirth and those with live births. For women with an NRT prescription, the average duration of NRT prescription was 2 weeks (interquartile range: 6 days to 2weeks), and 80% of NRT prescriptions were recorded within the first two trimesters.

**Table 1.** Baseline Characteristics of the Study Population by Outcome

	All pregnancies (*N* = 220 630)		Pregnancies ending in live birth (*N* = 219 825)		Pregnancies ending in stillbirth (*N* = 805)		*p* value
	*n*	%	*n*	%	*n*	%	
Age at conception							
** 15–19 years**	9731	4.4	9684	4.4	47	5.8	<.001
** 20–24 years**	32 585	14.8	32 453	14.8	132	16.4	
** 25–29 years**	58 649	26.6	58 459	26.6	190	23.6	
** 30–34 years**	70 605	32.0	70 393	32.0	212	26.3	
** 35–39 years**	40 210	18.2	40 043	18.2	167	20.7	
** 40–44 years**	8406	3.8	8352	3.8	54	6.7	
** 45–49 years**	444	0.2	441	0.2	3	0.4	
Townsend score in quintiles							
** Quintile 1 (least deprived**)	48 205	21.8	48 071	21.9	134	16.6	.001
** Quintile 2**	41 210	18.7	41 064	18.7	146	18.1	
** Quintile 3**	43 584	19.8	43 411	19.7	173	21.5	
** Quintile 4**	40 455	18.3	40 300	18.3	155	19.3	
** Quintile 5 (most deprived**)	29 431	13.3	29 294	13.3	137	17.0	
** Missing**	17,745	8.0	17,685	8.0	60	7.5	
Preconception **b**ody **m**ass **i**ndex (kg/m^2^)							
** Normal (18.5–24.9**)	81 408	36.9	81 157	36.9	251	31.2	.001
** Underweight (<18.5**)	5445	2.5	5424	2.5	21	2.6	
** Overweight (25–29.9**)	39 959	18.1	39 796	18.1	163	20.2	
** Obese (≥30**)	28 308	12.8	28 174	12.8	134	16.6	
** Missing**	65 510	29.7	65 274	29.7	236	29.3	
Asthma	22 444	10.2	22 368	10.2	76	9.4	.491
Hypertension	6502	2.9	6472	2.9	30	3.7	.190
Diabetes	7076	3.2	7036	3.2	40	5.0	.004
Mental **i**llness	19 344	8.8	19 273	8.8	71	8.8	.958

Table 2 presents the characteristics of mothers according to each exposure group. Mothers in the smoking and NRT group were considerably younger compared with the control group. About half of the mothers in the NRT and smoking group belonged to the two most deprived quintiles compared with about a quarter in the control group. In addition, there was a higher proportion of mothers in the NRT and smoking groups with mental illness compared with the control group (20.0%, 16.0%, and 7.8%, respectively).

**Table 2.** Baseline Characteristics of the Study Population by Exposure Status

	All pregnancies (*N* = 220 630)		Control group (*N* = 197 002)		Smokers (*N* = 18 407)		NRT group (*N* = 5221)		*p* value
	*n*	%	*n*	%	*n*	%	*n*	%	
Age at conception									
** 15–19 years**	9731	4.4	6890	3.5	2384	13.0	457	8.8	<.001
** 20–24 years**	32 585	14.8	26 095	13.2	5211	28.3	1279	24.5	
** 25–29 years**	58 649	26.6	52 440	26.6	4755	25.8	1454	27.8	
** 30–34 years**	70 605	32.0	65 794	33.4	3636	19.8	1175	22.5	
** 35–39 years**	40 210	18.2	37 582	19.1	1931	10.5	697	13.3	
** 40–44 years**	8406	3.8	7797	4.0	456	2.5	153	2.9	
** 45–49 years**	444	0.2	404	0.2	34	0.2	6	0.1	
Townsend score in quintiles									
** Quintile 1 (least deprived**)	48 205	21.8	45 900	23.3	1792	9.7	513	9.8	<.001
** Quintile 2**	41 210	18.7	38 369	19.5	2195	11.9	646	12.4	
** Quintile 3**	43 584	19.8	39 024	19.8	3541	19.2	1019	19.5	
** Quintile 4**	40 455	18.3	34 236	17.4	4805	26.1	1414	27.1	
** Quintile 5 (most deprived**)	29 431	13.3	23 348	11.9	4816	26.2	1267	24.3	
** Missing**	17 745	8.0	16 125	8.2	1258	6.8	362	6.9	
Preconception **b**ody **m**ass **i**ndex (kg/m^2^)									
** Normal (18.5–24.9**)	81 408	36.9	74 752	37.9	5101	27.7	1555	29.8	<.001
** Underweight (<18.5**)	5445	2.5	4681	2.4	606	3.3	158	3.0	
** Overweight (25–29.9**)	39 959	18.1	36 446	18.5	2662	14.5	851	16.3	
** Obese (≥30**)	28 308	12.8	25 444	12.9	2214	12.0	650	12.4	
** Missing**	65 510	29.7	55 679	28.3	7824	42.5	2007	38.4	
Asthma	22 444	10.2	19 565	9.9	2227	12.1	652	12.5	<.001
Hypertension	6502	2.9	6028	3.1	368	2.0	106	2.0	<.001
Diabetes	7076	3.2	6518	3.3	418	2.3	140	2.7	<.001
Mental **i**llness	19 344	8.8	15 350	7.8	2952	16.0	1042	20.0	<.001

NRT = nicotine replacement therapy.

### Absolute and Relative Risks of Stillbirth

Table 3 presents the absolute and relative risks of stillbirth by each exposure group. The absolute risk of stillbirth in the NRT group and amongst smokers was 5/1000 births, compared with 3.5/1000 births in the control group. In the unadjusted analysis, NRT was associated with a 44% increase in the odds of stillbirth compared with the reference group that was not statistically significant (OR 1.47, 95% CI 0.97 to 2.14) while smoking was associated with a statistically significant 52% increase in the risk of stillbirth (OR 1.52, 95% CI 1.23 to 1.89). After adjusting for potential confounders including maternal age, socioeconomic status, pre-pregnancy body mass index, and diabetes, there was still no statistically significant increase in the risk of stillbirth in the NRT group in comparison with the control group (OR 1.35, 95% CI 0.91 to 2.00); however, smoking during pregnancy was still associated with a 41% statistically significant increased risk of stillbirth (OR 1.41, 95% CI 1.13 to 1.77). When the reference group was changed to smokers, there was still no statistically significant association between NRT and stillbirth (OR 0.95, 95% CI 0.62 to 1.48).

**Table 3.** Absolute and Relative Risks of Stillbirth for NRT and Smoking Groups Compared With Controls

	Absolute risk of stillbirth		Unadjusted OR (95% CI)	*p* value	Adjusted OR (95% CI)^a^	*p* value
	*n*	%				
Control **g**roup	683	0.35	Reference		Reference	
NRT group	26	0.50	1.44 (0.97 to 2.14)	.069	1.35 (0.91 to 2.00)	.139
Smokers	96	0.52	1.52 (1.23 to 1.89)	<.001	1.41 (1.13 to 1.77)	.003

CI = confidence interval; NRT = nicotine replacement therapy; OR = odds ratio.

^a^Adjusted for maternal age, socioeconomic status, pre-pregnancy body mass index, and diabetes.

## Discussion

### Main Findings

Using 220 630 singleton pregnancies, we found that the absolute risk of stillbirth was very similar between the NRT and smoker groups. Although the effect estimates for both NRT and smoking were very similar, we found no statistically significant association between NRT being prescribed during pregnancy and stillbirth. Women who smoked during pregnancy had a 41% increased risk of stillbirth compared with the control group, which included never-smokers and ex-smokers.

### Strengths and Limitations

This is the largest study to date to investigate the association between NRT prescribing in pregnant women and stillbirth. However, because of the low prescribing of NRT in pregnancy,^8^ an even larger sample size is required to assess the association with adequate power. Stillborn babies are usually not registered in primary care. Therefore, the ascertainment of stillbirth in this study is based on the documentation of such events in maternal primary care records. Approximately 97% of deliveries in England and Wales in 2011 took place in NHS hospitals, maternity units, and maternity wings,^34^ and all the delivery information recorded in inpatient data should, but may not always, be transferred into primary care records. Therefore, we may have missed cases of stillbirth. Nevertheless, the prevalence of stillbirth in this study was 3.6/1000 live and stillbirths, which is comparable to the national prevalence of 5.2/1000 births in the United Kingdom.^35^ This slightly lower rate of stillbirth in THIN compared with the national prevalence may be attributed to the slight under-representation of general practices from more socioeconomically deprived areas in THIN. The effects of smoking or NRT use during pregnancy on stillbirth may be mediated by outcomes such as congenital anomalies;^36^ however, comprehensive data on congenital anomalies in these stillbirths are not available as a very small proportion of congenital anomalies are diagnosed antenatally and it is not routine conduct a full autopsy after stillbirth for a full diagnosis of congenital anomalies. Furthermore, in our earlier work, we found no statistically significant increased risk for major congenital anomalies associated with NRT use.^9^

We used primary care records to ascertain NRT exposure which is a more objective measurement of drug prescribing and use during pregnancy than self-reports by mothers in other studies.^37,38^ We recognize, however, the inherent inaccuracy in ascertaining true drug exposure in observational studies. In the United Kingdom, pregnant women can access NRT in settings other than the GP practice, such as through NHS Stop Smoking Services for Pregnancy (SSSP), over-the-counter purchases in pharmacies, and off-the-shelf purchases in supermarkets. However, only 3% of pregnant women access an SSSP on average each year,^39,40^ and a survey of all SSSPs in England conducted between April 2010 and March 2011 reported that almost half of the NRT provided by these services was issued through GPs.^41^ Furthermore, the prevalence of medication use without prior consultation with a health professional is lower during pregnancy than when women are not pregnant;^42^ NRT packaging clearly instructs pregnant women to consult a doctor before using these, and in the United Kingdom, women are entitled to free NHS prescriptions during pregnancy.^43^ Therefore, we believe that self-purchase of NRT would be infrequent in this group and misclassification in the exposure measurement should be minimal; however, the true magnitude of exposure misclassification is potentially unquantifiable and therefore results should be interpreted with caution. Since 2010, in addition to its use for smoking cessation NRT has been indicated for harm reduction, such that smokers could use NRT to cut down on smoking. Nevertheless, this indication does not apply to pregnant women. Therefore, we believe that simultaneous use of NRT and cigarettes should be minimal. Measuring concurrent usage in an epidemiological study would only be possible with a detailed follow-up study that would need to have multiple prospective recordings of women’s smoking behavior and close behavior monitoring. Another potential concern is that prescriptions issued are not necessarily an indication of compliance to drug therapy, and some of the prescriptions issued may not have been redeemed. Similarly, smoking cessation does not necessarily follow being prescribed with NRT, and some women in receipt of prescriptions may have continued to smoke. However, a validation study comparing the recorded prescriptions for smoking cessation medications in THIN and the NHS dispensing data between January 2004 and December 2005 reported good comparability between the two data sources indicating that prescriptions recorded for smoking cessation medications in primary care data are collected by the patients.^44^ Furthermore, measuring actual drug consumption in any large population-based study is pragmatically difficult and is a limitation in previous studies as well.^37^

Accurate ascertainment of smoking status is also difficult, especially in pregnant women, due to the social stigma attached to smoking. However, all previous epidemiological studies investigating the association between maternal smoking and stillbirth have similarly relied on self-reported smoking status data, as biochemical validation of large samples is expensive and often practically prohibitive throughout pregnancy. In our study, smoking status was self-reported in primary care and recorded during pregnancy by doctors, nurses, or midwives. Smoking prevalence from THIN based on the QOF rules, using information recorded within 27 months before pregnancy, shows good agreement with smoking estimates from other national datasets including the Scottish Morbidity Record and the Child Health Systems Programme data.^17^ Self-reports of smoking habit to health professionals invested in the person’s clinical care have shown to be reasonably accurate compared with those in bespoke studies.^45^

### Interpretation in Light of Other Evidence

Our study found no statistically significant increased risk of stillbirth associated with NRT exposure during pregnancy. The study of 87 032 pregnancies from the DNBC had information on smoking and NRT use that was self-reported by women during an interview at approximately 17 weeks of gestation. For women who used NRT, there was no statistically significant increase in the risk of stillbirth (HR 0.57, 95% CI 0.28 to 1.16) compared with nonusers.^46^ Furthermore, compared with nonsmokers and non-NRT users (ie, controls), the HR for stillbirth among women who smoked and used NRT simultaneously was 0.83 (95% CI 0.34 to 2.00) and among nonsmokers who used NRT was 0.67 (95% CI 0.21 to 2.08). However, the nonsmoker group in their study included ex-smokers who quit before conception but also women who were smoking during pregnancy but not at the time of the interview, and therefore, it is quite difficult to exclusively differentiate between the two, which could potentially result in misclassification of the exposure. They also did not have information on smoking or NRT after the interview so this is also a potential source of exposure misclassification. Furthermore, the time periods to define stillbirth were considerably different between both the DNBC study and our study: the DNBC study classified stillbirth as any fetus that did not breathe or show any other sign of life at birth after a minimum of 20 weeks of gestation compared with 28 weeks in our study. The potential differences between the exposure window and definition of stillbirth and the small number of stillbirth cases in the NRT group (*n* = 8) in the DNBC study could potentially explain the difference in findings. Nevertheless, our results are also in line with the pooled estimates from a meta-analysis of randomized controlled trial data (risk ratio 1.24, 95% CI 0.54 to 2.84),^10^ again suggesting that the use of NRT does not significantly increase or reduce the risk of stillbirth.

While the effect estimates for NRT and smoking were very similar, we found a 41% statistically significant increase in the risk of stillbirth in smokers. Exposure misclassification or residual confounding could be potential explanations or it could be that nicotine whether in the form of NRT or cigarette has a very similar effect on the growing fetus. Nevertheless, the statistically significant association between smoking and stillbirth is consistent with the current literature. The DNBC study found the risk of stillbirth to be 46% higher in smokers (HR 1.46, 95% CI 1.17 to 1.82) compared with nonsmokers using smoking information from the first trimester.^46^ Similarly, another Danish study based on a cohort of 25 102 live born singleton children collected smoking data before 30 weeks of gestation and found the risk of stillbirth to be twice as high compared with nonsmokers.^47^ Other studies that assessed smoking at the end of pregnancy or where the exact time of smoking assessment was not specified found the risk of stillbirth in smokers to be between 34%^48^ and over twofold^1,24,47,49,50^ higher compared with nonsmokers.

Tobacco smoke contains many harmful chemicals including nicotine, carbon monoxide, and nitrogen oxides^51,52^ and reduces fetal oxygenation through increased blood levels of carboxyhaemoglobin and impairment of oxygen unloading.^47^ This, along with prostacyclin synthesis, increases vascular resistance and decreases fetal blood flow. All these effects combined with the postulated vasoconstriction through nicotine^47^ could collectively result in fetal growth restriction and placental complications, which are the most important causes of stillbirth.^53,54^ However, we found the effect estimates in the NRT and smoking group to be very similar and compared directly to maternal smoking the risk estimates for stillbirth were not higher in the NRT group. Another potential explanation for the similar effect estimates in both groups is that pregnant women in the sample may have used only short courses of NRT (~2 weeks); long-term adherence with NRT in pregnancy has been poor in other studies. Data from the Smoking, Nicotine, and Pregnancy trial also show that only 7.2% women in the NRT group and 2.8% in the placebo group continued to use NRT beyond 1 month.^11^ Therefore, although we ensured that women prescribed NRT had no subsequent records of smoking throughout pregnancy, it is possible that some women did recommence smoking but did not report it to their GP or midwife.

## Conclusion

Previous evidence on maternal NRT exposure during pregnancy and the risk of stillbirth is limited and inconclusive. In this study, with much larger numbers than others, we did not find a protective or harmful effect of NRT prescribed during pregnancy as part of routine clinical care, in relation to stillbirth. While there may be no true association between NRT exposure during pregnancy and stillbirth, the potential impacts of residual confounding, misclassification of the exposure, and limited power must be recognized. An even larger study with biochemically validated data on active and passive smoking exposures, including close monitoring of independent NRT use and concurrent use with smoking throughout the 9 months of pregnancy, is required to determine this more definitively. All data are anonymised, such that individual patients as well as the name and speciﬁc location of general practices cannot be identiﬁed by researchers. Ethical approval for this research was obtained from the South-East Multicentre Research Ethics Committee (SE-REC), reference 04/MRE01/9. All data are anonymised, such that individual patients as well as the name and speciﬁc location of general practices cannot be identiﬁed by researchers. Ethical approval for this research was obtained from the South-East Multicentre Research Ethics Committee (SE-REC), reference 04/MRE01/9.

## Funding

This article presents independent research funded by the NIHR under its Programme Grants for Applied Research Programme (reference RP-PG 0109-10020). The funders had no role in study design, data collection and analysis, decision to publish, or preparation of the manuscript.

## Ethical Approval

All data are anonymized such that individual patients, their names, and specific location of the general practice cannot be identified. Ethical approval for the analysis of THIN data was obtained from the National Health Service (NHS) Medical Research Ethics Committee (REC Ref. 04/MRE01/9).

## Declaration of Interests

*None declared.*
